# Partially replacing cornstarch in a high-concentrate diet with sucrose inhibited the ruminal *trans*-10 biohydrogenation pathway *in vitro* by changing populations of specific bacteria

**DOI:** 10.1186/s40104-015-0051-y

**Published:** 2015-12-24

**Authors:** Xiaoqin Sun, Yaping Wang, Bo Chen, Xin Zhao

**Affiliations:** College of Animal Science and Technology, Northwest A & F University, Yangling, Shaanxi 712100 People’s Republic of China; Department of Animal Science, McGill University, 21,111 Lakeshore, Ste. Anne de Bellevue, QC H9X 3V9 Canada

**Keywords:** Bacterial populations, Cornstarch, Rumen fermentation, Sucrose, *trans*-10 shift

## Abstract

**Background:**

The positive influence of replacing dietary starch with sugar on milk fat production has been proposed to be partially attributed to the inhibition of the rumen *trans*-10 biohydrogenation pathway. However, whether and how sucrose inhibits the rumen *trans*-10 biohydrogenation pathway remains elusive.

**Results:**

A batch *in vitro* incubation system was used to evaluate effects of replacing cornstarch in a high-concentrate diet (forage to concentrate ratio = 40:60) with 0 (control), 3, 6 and 9 % of sucrose on rumen fermentation pattern, fatty acid (FA) biohydrogenation pathways and bacterial populations relating to *trans*-11 to *trans*-10 biohydrogenation pathways. Replacing dietary cornstarch with sucrose did not alter rumen pH or concentrations of total volatile fatty acids (VFA) in comparison with the control but significantly influenced the profiles of individual VFA. The molar proportions of butyrate and valerate were linearly increased, while that of acetate was quadratically decreased and those of propionate, isobutyrate and isovalerate were linearly decreased with increasing concentrations of sucrose in the diet. Furthermore, replacing cornstarch with sucrose led to a linear decrease in C18:1 *trans*-10, linear increases in the proportions of C18:1 *trans*-11, C18:2n-6 and the ratio of *trans*-11 to *trans*-10, and linear decreases in biohydrogenation of C18:2n-6 and C18:3n-3. The abundance of *Butyrivibrio fibrisolvens*, a butyrate and CLA *cis*-9, *trans*-11 producer, was increased with the increasing inclusion of sucrose in the diet, while the population of *Megasphaera elsdenii*, a CLA *trans*-10, *cis*-12 producer, was significantly decreased by all levels of sucrose replacements.

**Conclusions:**

These results indicate that replacing starch in a high-concentrate diet with sucrose increased butyrate production and inhibited the rumen *trans*-10 biohydrogenation pathway, which was at least partially due to increased abundance of *Butyrivibrio fibrisolvens* and decreased abundance of *Megasphaera elsdenii*.

## Background

Feeding high-concentrate diets is a commonly used strategy to increase energy intake and support high milk yield in the intensive dairy production system. However, it is also a well-known factor inducing rumen acidosis and milk fat depression (MFD). In order to maintain animal energy intake and simultaneously reduce the risks of rumen acidosis and MFD, sugar has been used as an alternative energy source. Partially replacing dietary starch or corn grain with sugar did not decrease rumen pH in several studies [1, 2] or even increased rumen pH in others [3]. More importantly, feeding sugar improved milk fat content and milk fat yield [3, 4]. However, the action mechanism for the observed results is not well understood. For example, it is not known why feeding sugar potentially increases rumen pH despite its rapid rumen fermentation relative to starch. One plausible reason could be increased production of butyrate by sugar fermentation. However, effects of sugar addition on profiles of rumen volatile fatty acids (VFA) have been considerably variable. Most *in vitro* studies reported increased production of butyrate by including sugar in the diet [2, 5, 6]. As for *in vivo* studies, some reported increased butyrate concentrations by sugar-feeding [1, 3], while others showed no influence on concentrations of acetate, propionate, butyrate [7] or all individual VFA [4, 8]. Concentrations of VFA in the rumen reflect a balance between acid production and disappearance. Actual production of butyrate in the rumen is likely underestimated under *in vivo* conditions [9], due to the faster absorption rate of butyrate than those of acetate and propionate. Thus, *in vitro* studies might better reflect the actual fermentation characteristics of sugar-containing diets.

The positive influence of sugar on milk fat has been proposed to be partially attributed to inhibition of the rumen *trans*-10 biohydrogenation pathway [3, 9]. Occurrence of MFD induced by high-concentrate diets is known to be associated with the shift of rumen biohydrogenation pathways from *trans*-11 to *trans*-10 [10], which produce *trans*-10 fatty acids (FA), specifically C18:1 *trans*-10 and CLA *trans*-10,*cis*-12 at the expense of C18:1 *trans*-11 and CLA *cis*-9,*trans*-11. However, direct investigation of sugar on rumen biohydrogenation of FA is limited.

Variations in rumen VFA production and biohydrogenation of FA are associated directly with rumen microflora. However, influence of sugar on rumen microbial populations has not been extensively studied. The lactate-producing (*Streptococcus bovis*, *S. bovis*) or utilizing bacteria (*Megasphaera elsdenii*, *M. elsdenii*; *Propionibacterium acnes*, *P. acnes*) that proliferate in the rumen of animals receiving high-concentrate diets are major CLA *trans*-10, *cis*-12 producers [11–13]. In contrast, the well known CLA *cis*-9,*trans*-11 producer [14, 15], *Butyrivibrio fibrisolvens* (*B. fibrisolvens*), is very sensitive to high-starch diets and its population in the rumen declined considerably with concentrate feeding [16]. It is likely that replacing dietary starch with sugar could inhibit the proliferating of the CLA *trans*-10, *cis-*12 producers, reducing the production of the antilipogenic *trans*- FA and consequently improving milk fat synthesis. Unfortunately, no study, to the best of our knowledge, has investigated the effect of sugar feeding on rumen microbial community, especially the species related to rumen *trans*-11 to *trans*-10 biohydrogenation pathways.

The objective of the current *in vitro* study, therefore, was to evaluate the responses of partially replacing cornstarch in a high-concentrate diet with sucrose on rumen fermentation, biohydrogenation of FA and bacterial populations related to the rumen *trans*-10 shift.

## Methods

### Experimental design

A completely random design was used in an *in vitro* batch incubation system. Cornstarch in a basal diet (Table 1) with a concentrate to forage rate of 60:40 was replaced by four levels of sucrose: 0 (control), 3, 6 and 9 % of dry matter (DM).

**Table 1:** Ingredients and chemical composition of the basal diet (DM basis)

Ingredient	Content, %
Alfalfa hay	14.50
Corn silage	25.50
Corn	28.20
Soybean meal	18.60
Cornstarch	9.00
Corn gluten meal	2.50
Sodium bicarbonate	0.40
Mineral and vitamin premix ^1^	0.20
Dicalcium phosphate	0.20
Limestone	0.46
Salt	0.44
Chemical composition (% DM)	
Organic matter	94.3
Crude protein	15.5
Non fiber carbohydrates ^2^	49.9
Neutral detergent fibre	25.1
Acid detergent fibre	16.6
Starch	38.7
Sugar	3.96

^1^Providing Mn 63 mg, Zn 4,640 mg, Fe 972 mg, Cu 265 mg, Se 155 mg, I 251 mg, Co 57 mg, nicotinic acid 450 mg, VE 800 mg, VD 450 KIU and VA 1,860 KIU per kilogram

^2^Calculated by 100 – (% crude protein + % ether extract + % neutral detergent fibre)

### *In vitro* incubation and sampling

The batch incubation was carried out with 1.0 g of the four experimental diets with 30 mL of buffer [17] and 30 mL of rumen fluid at 39 °C, shaking at 165 rpm. The rumen fluid was collected from four ruminally-cannulated dairy goats before morning feeding. The whole procedure was kept under strict anaerobic conditions by gassing the buffer with CO_2_ and using resazurin as a redox indicator, flushing the rumen fluid during handling with CO_2,_ and gassing the headspace of incubation bottles before sealing with CO_2_ as well. Samples from each treatment at 0 h incubation were frozen and freeze-dried for FA analyses and biohydrogenation calculation. The incubation periods were set for 6, 12 and 24 h, four replicates per treatment at each time point. At the end of 6 h incubation, pH was measured immediately and fermentation was stopped on ice. Samples for VFA (3 mL), ammonia-N (2 mL) and rumen bacterial populations (1 mL) were individually collected. One mL of 25 % meta phosphoric acid was added to VFA samples, while 2 to 3 drops of 25 % sulphuric acid was added to ammonia-N samples. These samples were stored at −20 °C until analyses. Samples for bacterial determination were centrifuged at 20,000 × g (4 °C) for 5 min immediately after collection and the pellets were stored at −80 °C. Residues in the incubated bottles were frozen, freeze-dried, weighed and stored at −20 °C for FA analyses. After 12 and 24 h incubation, samples were collected for rumen bacterial population determination.

### Chemical analysis

Nitrogen, DM, acid detergent fibre, ether extract and ash in the basal diet were analyzed according to the methods described by AOAC [18]. Crude protein was determined by multiplying nitrogen by 6.25. Neutral detergent fibre was assayed with a heat stable α-amylase and sodium sulfite according to Van Soest et al. [19], expressed inclusive of residual ash. Non structural carbohydrate was calculated by 100 – (% crude protein + % ether extract + % neutral detergent fibre). Starch was analyzed according to the enzymatic-colorimetric method [20]. Sugar was extracted with ethanol/water solution (v/v = 80:20) and followed by the phenol-sulphuric acid assay [21].

Samples for VFA and ammonia-N analyses were thawed, centrifuged at 20,000 × g for 20 min at 4 °C, and supernatant filtered through a 0.45 μm syringe filter were used for analysis. The pellets were freeze-dried and weighed for biohydrogenation estimation. VFA was determined by gas chromatography (GC-7890, Agilent Technologies, Hewlett Packard Co., Avondale, PA) fitted with a flame-ionization detector using a 30 m × 0.25 mm × 0.33 μm fused silica column (AE-FFAP; Atech Technologies Co. Ltd., Taipei, Taiwan, China) according to Khorasani et al. [22]. Ammonia-N was determined by colorimetry as described by Fawcett and Scott [23].

The composition of FA was analyzed as described by Sun and Gibbs [24]. Briefly, the freeze-dried samples were directly methylated with 4 mL of 0.5 mol/L NaOH/methanol (15 min at 50 °C) followed by 4 mL of 5 % HCl/methanol (1 h at 50 °C), extracted with 2 mL of heptane and then introduced to the GC equipped with a fused silica capillary column (SP-2560, 100 m × 0.25 mm × 0.2 μm; Supelco Inc., Bellefonte, PA). Nitrogen was used as the carrier gas. The split ratio was 50:1, and the injector and detector were held at 250 °C. The temperature gradient was 166 °C for 39 min, increased by 10.0 °C/min to 240 °C, held for 10 min, increased by 3.0 °C/min to 245 °C, and held for 10 min. C19:0 was used as an internal standard, and ME 61, ME93, BR_2_, BR_3_, CLA *cis*-9, *trans*-11 and CLA *trans*-10, *cis*-12 methyl esters were used as external standards (Larodan Fine Chemicals AB, Malmo, Sweden). Peaks of four C18:1 (*trans*-4, *trans*-5, *trans*-6/7/8, *trans*-10) and six C18:2 (*trans*-9, *trans*-12, *cis*-9, *trans*-13, *trans*-8, *cis*-13, *cis*-9, *trans*-12, *trans*-9, *cis*-12, *trans*-11, *cis*-15) isomers without commercial external standards were identified by the order of elution with respect to those FA with external standards and interpolation from the peaks reported [25].

Total microbial genomic DNA was extracted according to Kittelmann et al. [26]. Briefly, microbial cells were disrupted by bead-beating and microbial genomic DNA was extracted by phenol-chloroform extraction. DNA was subsequently precipitated by adding 1/10 volume of 3 mol/L sodium acetate (pH 5.2) and 2 volumes of 70 % ice-cold ethanol. The precipitate was then resuspended in 100 μL of an elution buffer (10 mmol/L Tris–HCl, pH 8.5). RNA was eliminated by adding 10 mg/mL of RNase A, and DNA was subsequently cleaned up using the EasyPure PCR purification kit (*Trans*gen, China) and eluted in 50 μL EB. The concentration and purity of the extracted DNA were determined by a NanoDrop spectrophotometer (Thermo Scientific, USA).

Quantitative real-time PCR was performed on a CFX96 Real-Time PCR Detection System (Bio-Rad, USA) to investigate the abundance of *B. fibrisolvens*, *S. bovis*, *M. elsdenii* and *P. acnes* as described by Li et al. [27]. Briefly, a 25-μL PCR mixture was prepared using 12.5 μL of SYBR Premix Ex TaqII (TAKARA, China), 1 μL of forward primer (10 μmol/L), 1 μL of reverse primer (10 μmol/L), 1 μL of DNA, and 9.5 μL of double-distilled water. The thermal cycling parameters were programmed at 95 °C for 30 s for initial denaturation, 40 cycles of 95 °C for 5 s, and 60 °C for 30 s for primer annealing and product elongation. Fluorescence detection was performed at the end of each extension step. The 16S rRNA gene-targeted primer sets are listed in Table 2. Samples were run as triplicate. Amplicon specificity was determined via melt curve analysis of PCR end products by raising the temperature at the rate of 1 °C per 30 s from 60 to 95 °C. The population sizes of specific bacterial groups were expressed as a percentage relative to the abundance of the general bacterial 16S rRNA gene.

**Table 2:** Primers used for real-time PCR

Target species	Primer	Sequence (5′ → 3′)	Reference
General bacteria	Forward	CGGCAACGAGCGCAACCC	McSweeney and Denman [41]
	Reverse	CCATTGTAGCACGTGTGTAGCC	
*Butyrivibrio fibrisolvens*	Forward	ACCGCATAAGCGCACGGA	Stevenson and Weimer [42]
	Reverse	CGGGTCCATCTTGTACCGATAAAT	
*Streptococcus bovis*	Forward	TTCCTAGAGATAGGAAGTTTCTTCGG	Stevenson and Weimer [42]
	Reverse	ATGATGGCAACTAACAATAGGGGT	
*Megasphaera elsdenii*	Forward	AGATGGGGACAACAGCTGGA	Stevenson and Weimer [42]
	Reverse	CGAAAGCTCCGAAGAGCCT	
*Propionibacterium acnes*	Forward	GGGTTGTAAACCGCTTTCGCCTG	Shingfield et al. [43]
	Reverse	TGCTTTCGATACGGGTTGAC	

### Calculation and statistical analysis

Biohydrogenation of C18:2n-6 and C18:3n-3 was estimated based on disappearance of these fatty acids from 0 h incubation to the end of incubation. Total weights of the residues after incubation were estimated by summing up the freeze-dried weights of contents in the incubation bottles and those pellets from VFA and ammonia-N samples.

Data were analyzed using One-way ANOVA of SPSS17.0 (SPSS software for Windows, release 17.0, SPSS Inc., Chicago, IL). Tukey’s test was used for multiple comparisons, and orthogonal polynomials were used to test linear and quadratic responses for increasing levels of sucrose in the diet. Significance was declared at *P* < 0.05.

## Results

### Rumen fermentation

As shown in Table 3, replacing dietary cornstarch with 3 to 9 % of sucrose had no significant influence on rumen pH (*P* = 0.971) and total VFA concentrations (*P* = 0.056) after 6 h *in vitro* incubation. However, the profiles of individual VFA were affected significantly (*P* < 0.05) by sucrose treatment. The molar proportion of acetate decreased quadratically (*P* < 0.001), while those of propionate, isobutyrate and isovalerate decreased linearly (*P* < 0.001) as the inclusion of sucrose increased. The molar proportion of butyrate increased quadratically (*P* < 0.001) with increasing sucrose addition, while the molar proportion of valerate increased linearly (*P* = 0.002). Concentrations of ammonia-N decreased linearly (*P* < 0.001) with the inclusion of sucrose.

**Table 3:** Rumen pH, volatile fatty acid (VFA) profiles and ammonia-N concentration after 6 h *in vitro* incubation

Item	Sucrose level				SEM^1^	*P*-value^2^		
	0	3 %	6 %	9 %		T	L	Q
Rumen pH	6.17	6.17	6.18	6.19	0.014	0.971	0.641	1.000
Total VFA*,* mmol/L	73.8	74.8	75.9	76.5	0.391	0.056	0.009	0.738
Individual VFA (mol/100 mol)								
Acetate	63.0^a^	62.8^b^	62.7^b^	63.1^a^	0.044	0.003	0.380	<0.001
Propionate	20.4^a^	20.1^b^	19.9^bc^	19.7^c^	0.074	0.001	<0.001	0.324
Butyrate	12.2^c^	12.7^b^	13.1^a^	12.9^ab^	0.090	<0.001	<0.001	<0.001
Isobutyrate	1.16^a^	1.14^b^	1.11^c^	1.08^d^	0.009	<0.001	<0.001	0.715
Valerate	1.25^b^	1.29^ab^	1.30^a^	1.31^a^	0.008	0.015	0.002	0.457
Isovalerate	2.07^a^	2.01^b^	1.95^c^	1.89^d^	0.018	<0.001	<0.001	0.804
A:P ratio^3^	3.09^c^	3.12^bc^	3.16^ab^	3.19^a^	0.012	0.002	<0.001	0.987
Ammonia, mg/dL	28.9^a^	27.5^b^	26.8^b^	26.6^b^	0.291	0.005	<0.001	0.114

Note: Means within the same row with different superscripts differ significantly (*P* < 0.05)

^1^ SEM = standard error of mean

^2^ Probability of a significant effect of treatment (T), or linear (L) and quadratic (Q) orthogonal contrasts for dietary sucrose levels

^3^ A:P ratio = acetate to propionate ratio

### Fatty acids composition and biohydrogenation

Effects of replacing dietary cornstarch with sucrose on FA composition and biohydrogenation after 6 h *in vitro* incubation were presented in Tables 4 and 5. Sucrose inclusion had no effect (*P* > 0.05) on the proportions of the medium-chain FA and long-chain saturated FA (Table 4). However, the proportions of some C18 FA isomers were influenced (*P* < 0.05) (Table 5). For C18:1 isomers, the proportions of C18:1 *trans*-5, C18:1 *trans*-10 and C18:1 *trans*-11 were affected significantly (*P* < 0.05) by dietary treatments. C18:1 *trans*-10 decreased linearly (P <0.05) while C18:1 *trans*-11 increased linearly (*P* < 0.05) with the increasing inclusion of sucrose. For the two major dietary poly-unsaturated FA, the proportion of C18:2 n-6 was influenced (*P* < 0.05) by inclusion of sucrose, while proportion of C18:3 n-3 was not affected (*P* > 0.05) by the dietary treatment. The ratio of *trans*-11 to *trans*-10 was linearly increased (*P* > 0.05) by increasing inclusion of sucrose in the diet. Biohydrogenation of C18:2 n-6 and C18:3 n-3 both decreased linearly (*P* < 0.05) by increasing inclusion of sucrose.

**Table 4:** Composition of the medium- and long-chain fatty acid (FA) after 6 h *in vitro* incubation

Fatty acid, % of total FA	Sucrose level				SEM ^1^	*P*-value^2^		
	0	3 %	6 %	9 %		T	L	Q
C12:0	0.48	0.58	0.42	0.67	0.058	0.507	0.456	0.563
C13:0 anteiso	0.36	0.25	0.34	0.29	0.024	0.409	0.569	0.622
C13:0	0.21	0.18	0.26	0.23	0.015	0.324	0.321	0.970
C14:0 iso	0.71	0.57	0.56	0.69	0.033	0.214	0.825	0.044
C14:0	2.62	2.28	2.19	2.62	0.090	0.218	0.879	0.044
C14:1 *trans*-9	2.21	1.83	2.12	2.19	0.087	0.367	0.783	0.209
C15:0 anteiso	3.12	2.53	2.33	3.15	0.167	0.228	0.946	0.047
C14:1 *cis*-9	1.85	1.61	1.42	1.65	0.073	0.271	0.219	0.122
C15:0	1.23	1.02	1.11	1.40	0.078	0.333	0.375	0.124
C16:0 iso	1.22	1.04	1.01	1.12	0.040	0.126	0.785	0.022
C16:0	27.6	25.1	25.8	26.1	0.492	0.294	0.366	0.158
C16:1 *trans*-9	0.53	0.49	0.46	0.48	0.021	0.771	0.395	0.551
C17:0 anteiso	1.47	1.24	1.15	1.50	0.073	0.271	0.982	0.060
C16:1*cis*-9	0.69	068	0.63	0.80	0.034	0.391	0.358	0.218
C17:0	0.58	0.63	0.53	0.61	0.018	0.251	0.958	0.630
C18:0 iso	0.05	0.06	0.12	0.11	0.015	0.261	0.077	0.896
C20:0	0.45	0.53	0.47	0.55	0.021	0.229	0.151	1.000
C20:1 *trans*-11	0.19	0.20	0.21	0.20	0.007	0.771	0.576	0.396
C22:1 *trans*-13	0.04	0.05	0.05	0.05	0.004	0.470	0.225	0.526
C24:0	0.36	0.60	0.50	0.65	0.051	0.176	0.089	0.648
C26:0	0.13	0.18	0.14	0.20	0.020	0.632	0.388	0.909

^1^ *SEM* standard error of mean

^2^ Probability of a significant effect of treatment (T), or linear (L) and quadratic (Q) orthogonal contrasts for dietary sucrose levels

**Table 5:** C18 fatty acids (FA) composition and biohydrogenation of C18:2 n-6 and C18:3 n-3 after 6 h *in vitro* incubation

Fatty acid, % of total FA	Sucrose level				SEM^1^	*P*-value^2^		
	0	3 %	6 %	9 %		T	L	Q
C18:0	22.6	23.2	19.3	17.7	1.146	0.252	0.071	0.620
C18:1 *trans*-4	0.13	0.20	0.17	0.18	0.011	0.150	0.204	0.197
C18:1 *trans*-5	0.17^a^	0.11^b^	0.15^a^	0.14^ab^	0.008	0.017	0.256	0.064
C18:1 *tans*-6/7/8	3.79	3.84	3.59	3.29	0.135	0.475	0.171	0.531
C18:1 *trans*-9	0.14	0.11	0.20	0.14	0.020	0.567	0.636	0.799
C18:1 *trans*-10	0.38^a^	0.34^a^	0.24^b^	0.24^b^	0.021	0.011	0.002	0.554
C18:1 *trans*-11	8.70^b^	8.91^b^	10.1^a^	10.5^a^	0.107	0.040	0.021	0.549
C18:1 *cis*-9	1.48	1.58	1.75	1.62	0.061	0.565	0.303	0.381
C18:1 *cis*-6	0.26	0.25	0.22	0.20	0.020	0.702	0.259	0.997
C18:1 *cis*-11	0.16	0.15	0.081	0.16	0.123	0.093	0.491	0.079
C18:2 *trans*-9,*trans*-12	0.11	0.21	0.15	0.18	0.020	0.313	0.386	0.417
C18:2 *cis*-9,*trans*-13	0.058	0.048	0.061	0.062	0.004	0.699	0.542	0.579
C18:2 *trans*-8,*cis*-13	0.63	0.61	0.60	0.69	0.022	0.435	0.346	0.210
C18:2 *cis*-9,*trans*-12	0.064	0.058	0.061	0.078	0.004	0.303	0.209	0.168
C18:2 *trans*-9,*cis*-12	0.036	0.033	0.045	0.032	0.004	0.709	0.987	0.556
C18:2 *trans*-11,*cis*-15	0.14	0.13	0.12	0.13	0.011	0.962	0.922	0.641
C18:2 n-6	11.0^b^	13.6^ab^	16.4^a^	14.5^ab^	0.707	0.045	0.021	0.072
C18:3 n-3	2.34	3.00	3.11	2.75	0.114	0.063	0.131	0.021
CLA *cis*-9,*trans*-11	0.028	0.043	0.026	0.036	0.004	0.588	0.871	0.811
CLA *trans*-10,*cis*-12	0.036	0.033	0.032	0.029	0.003	0.894	0.473	0.985
*Trans*-11/*trans*-10^3^	19.1^c^	21.2^bc^	33.7^ab^	40.0^a^	2.959	0.012	0.002	0.609
Biohydrogenation (%)								
C18:2n-6	58.5^a^	43.8^b^	36.2^b^	36.3^b^	3.151	0.008	0.002	0.081
C18:3n-3	30.9^a^	25.8^ab^	17.4^c^	19.1^bc^	1.790	0.015	0.046	0.253

Note: Means within the same row with different superscripts differ significantly (*P* < 0.05)

^1^ SEM = standard error of mean

^2^ Probability of a significant effect of treatment (T), or linear (L) and quadratic (Q) orthogonal contrasts for dietary sucrose levels

^3^ *Tans-*11/*trans*-10 = ratio of total *trans*-11 C18 isomers (C18:1 *trans*-11 + CLA *cis*-9,*trans*-11) to *trans*-10 isomers (C18:1 *trans*-10 + CLA *trans*-10, *cis*-12)

### Bacterial populations

Replacing dietary cornstarch with sucrose did not influence (*P* > 0.05) the numbers of *B. fibrisolvens*, *M. elsdenii*, *S. bovis* or *P. acnes* after 6 and 12 h incubations (Table 6). Results from 24 h incubation indicated that sucrose inclusion linearly increased (*P* = 0.005) the abundance of *B. fibrisolvens,* whose numbers were greater (*P* < 0.05) in the 6 and 9 % sucrose treatments in comparison with the control and the 3 % treatment. The numbers of *M. elsdenii* after 24 h incubation were lower (*P* < 0.05) in three sucrose treatments compared to the control diet, however, no difference (*P* > 0.05) was observed among these treatments. The 3 % sucrose treatment had greater (*P* < 0.05) *S. bovis* populations than other treatments after 24 h incubation. However, the numbers of *P. acnes* were not affected (*P* = 0.227) by sucrose treatments even after 24 h incubation. The changing patterns of the measured bacterial groups were similar among 4 treatment groups, with the numbers of *B. fibrisolvens*, *S. bovis* and *P. acnes* increased and the number of *M. elsdenii* decreased over the time. However, sucrose inclusion caused larger changes than the control treatment.

**Table 6:** Rumen bacterial populations (% of total bacteria) after 6, 12 and 24 h *in vitro* incubation

Bacteria^1^	Incubation time	Sucrose level				SEM ^2^	*P*-value ^3^		
		0	3 %	6 %	9 %		T	L	Q
*B. fibrisolvens*	6 h	0.0013	0.0015	0.0018	0.0013	0.0007	0.063	0.580	0.018
	12 h	0.0016	0.0022	0.0023	0.0025	0.0001	0.142	0.033	0.480
	24 h	0.0017^c^	0.0020^bc^	0.0029^a^	0.0025^ab^	0.0002	0.008	0.005	0.110
*M. elsdenii*	6 h	0.041	0.041	0.035	0.044	0.0044	0.927	0.940	0.683
	12 h	0.037	0.025	0.035	0.043	0.0039	0.481	0.415	0.249
	24 h	0.034^a^	0.015^b^	0.021^b^	0.018^b^	0.0026	0.036	0.049	0.075
*S. bovis*	6 h	0.061	0.060	0.038	0.044	0.0121	0.898	0.537	0.886
	12 h	0.090	0.099	0.080	0.100	0.0057	0.718	0.756	0.548
	24 h	0.080^b^	0.18^a^	0.090^b^	0.11^b^	0.0139	0.016	0.848	0.062
*P. acnes*	6 h	0.029	0.035	0.050	0.054	0.0047	0.190	0.039	0.956
	12 h	0.073	0.074	0.061	0.069	0.0046	0.776	0.561	0.705
	24 h	0.080	0.103	0.077	0.087	0.0049	0.227	0.930	0.463

Note: Means within the same row with different superscripts differ significantly (*P* < 0.05)

^1^ *B. fibrisolvens* = *Butyrivibrio fibrisolvens*, *S. bovis* = *Streptococcus bovis*, *M. elsdenii* = *Megasphaera elsdenii*, *P. acnes* = *Propionibacterium acnes*

^2^ SEM = standard error of mean

^3^ Probability of a significant effect of treatment (T), or linear (L) and quadratic (Q) orthogonal contrasts for dietary sucrose levels

## Discussions

Results of the current study showed significant influences on all individual VFA by partially replacing dietary cornstarch with sucrose, with the molar proportions of butyrate and valerate were significantly increased (*P* < 0.05), while those of acetate, propionate, isobutyrate and isovalerate were significantly decreased (*P* < 0.01). This variation pattern was quite similar to the previous *in vitro* [2, 6] and *in vivo* [28] studies, except that the molar proportion of propionate was not affected in the study of Ribeiro et al. [6], while valerate was linearly reduced with the increasing level of dietary sucrose in the study of Vallimont [2]. The increased molar proportion of butyrate observed in the present study was consistent to the earlier *in vitro* [2, 6] and *in vivo* [3, 29] studies, confirming that fermentation of sucrose in place of starch increased butyrate production. Ribeiro et al. [6] suggested that the greater butyrate concentration could be attributed to a change in fermentation pathways to accommodate the higher flux of hydrogen from the rapidly fermented sugar source or the synthesis of this acid from lactate by *M. elsdenii*. In our study, the numbers of *M. elsdenii* were lower (*P* < 0.05) in three sucrose treatments after 24 h incubation compared to the control diet and it seems that *M. elsdenii* was not responsible for increased butyrate production. Instead, abundance of *B. fibrisolvens* increased after 24 h incubation, which might be at least partially responsible for increased butyrate production. Doubling ruminal butyrate concentrations by ruminal infusion of butyrate increased the relative abundance of *B. fibrisolvens* by 65 % [30]. In addition, oral administration of *B. fibrosolvens* in mice resulted in a significant increase in the rate of fecal butyrate production [31]. Butyrate is one of the major precursors of milk fat and increase of this acid may help explain the increased milk fat production observed in sugar feeding studies in vivo [3, 8].

The reduced production of branched-chain VFA (isobutyrate and isovalerate) found in the present study was consistent with the earlier studies [1, 6, 7], suggesting the deamination and decarboxylation of branched-chain amino acid formation system might be reduced by sucrose fermentation, because this is the producing pathway of rumen branched-chain VFA [32]. Besides, *M. elsdenii* were capable of deaminating branched-chain amino acid and producing branched-chain VFA [33], thus, the decreased branched-chain VFA proportion observed by sugar fermentation might be partially due to reduction in the population of *M. elsdenii* in the present study.

The unaffected (*P* > 0.05) pH and total VFA concentrations (Table 3) observed in the present study were consistent with the earlier findings based on sucrose replacing for starch [1, 2] or supplementing to alfalfa hay [6]. Our results support the suggestion that the rapid disappearance of sugar *per se* does not necessarily mean extensive fermentation, leading to increased acid production and low rumen pH [9]. To our knowledge, studies [29, 34] that reported decreased rumen pH by sucrose inclusion in the diets were not starch replacing experiments, and reasons for reduced rumen pH observed in those studies might be attributed to either extremely low-forage rations (only 10 % of DM) [34], or extremely high sucrose amount (approximately 20 % of DM) dosed to the rumen [29]. Thus, the present results indicated that replacing dietary cornstarch in a high-concentrate diet with up to 9 % of sucrose might not increase the risk of rumen acidosis. Decreased proton production by increased butyrate and valerate production and less carbon provided by sucrose fermentation compared to starch [35] could be the most possible explanations for the unaffected pH observed in the present study. As 1 mole of hexose ferments to 1 mole of butyrate or valerate, or to 2 moles of propionate or acetate [36], the greater butyrate and valerate production in the rumen from feeding sugar would decrease proton production per unit of organic matter fermented in the rumen compared with acetate or propionate production.

To our knowledge, this is the first study investigating the effects of partially replacing dietary starch with sucrose on the rumen *trans*-10 shift with a high-concentrate diet. Results of the current study demonstrated that replacing cornstarch in a high-concentrate diet with sucrose inhibited the rumen *trans*-10 shift by reducing C18:1 *trans*-10 production and increasing C18:1 *trans*-11 production. The variations in these two C18:1 *trans* isomers occurred in the absence of changes in their major C18:2 precursors, CLA *trans*-10, *cis*-12 and CLA *cis*-9, *trans*-11. The *trans*-11 to *trans*-10 ratio in rumen fluid has been considered as an indicator of changed rumen microbial populations [37]. However, although the ratio of *trans*-11 to *trans*-10 in the present study increased linearly from 19.1 to 40.0 with the increasing inclusion of sucrose in the diet after 6 h *in vitro* incubation, we failed to detect any statistical difference in the relative abundance of the four groups of bacteria after 6 or 12 h of incubation. Results from 24 h incubation showed that replacing cornstarch for sucrose substantially increased the populations of rumen CLA *cis*-9,*trans*-11 producing bacteria (*B. fibrisolvens*) and reduced numbers of one species (*M. elsdenil*) of the reported CLA *trans*-10, *cis*-12 producers. These results were in accordance with observed variations in the proportions of C18:1 *trans*-11 and C18:1 *trans*-10, indicating that the inhibition of rumen *trans*-10 shift by sucrose might be mainly related to a shift of these bacteria. It has been suggested that *trans*-10 shift could result from a dysbiosis in the rumen in favor of *trans*-10-producing bacteria at the expense of those producing *trans*-11 or a modification of bacterial activities [38]. Thus, the change of rumen bacteria by sucrose addition could be due to higher activities at the beginning and higher bacterial numbers later.

As a major cellulolytic microorganism and butyrate producer in the rumen, *B. fibrisolvens* has a high affinity toward maltose and sucrose utilization [39], which could be a possible explanation for the increased number of this bacterium by sucrose inclusion in the diet. However, *B. fibrisolvens* is also a major microbe related to rumen biohydrogenation [40], and the increase of its population theoretically means greater biohydrogenation of dietary unsaturated FA. However, biohydrogenation of C18:2 n-6 and C18:3 n-3 in the present study decreased linearly with increasing sucrose inclusion in the diet. Similar observation was also reported when sucrose was added to alfalfa hay [6]. Why biohydrogenation of C18:2 n-6 and C18:3 n-3 did not increase in this study warrants further investigation.

## Conclusions

Replacing cornstarch in a high-concentrate diet with sucrose *in vitro* did not alter rumen pH and total VFA concentration, but increased molar proportions of butyrate and valerate. Sucrose inclusion in the diet inhibited the rumen *trans*-10 biohydrogenation pathway as evidenced by decreased proportion of C18:1 *trans*-10 and increased proportion of C18:1 *trans*-11. Biohydrogenation of C18:2n-6 and C18:3n-3 was decreased by sucrose inclusion. These changes may be at least partially due to increased abundance of *B. fibrisolvens*, and decreased abundance of *M. elsdenii*.

## Abbreviations

CLA: conjugated linoleic acid
DM: dry matter
FA: fatty acids
MFD: milk fat depression
VFA: volatile fatty acid
